# Ilexonin A Promotes Neuronal Proliferation and Regeneration via Activation of the Canonical Wnt Signaling Pathway after Cerebral Ischemia Reperfusion in Rats

**DOI:** 10.1155/2016/9753189

**Published:** 2016-01-18

**Authors:** Bi-Qin Zhang, Guan-Yi Zheng, Yu Han, Xiao-Dong Chen, Qiong Jiang

**Affiliations:** ^1^The Affiliated Union Hospital of Fujian Medical University, Fuzhou 350001, China; ^2^Huai'an First People Hospital, Huai'an 223300, China

## Abstract

*Aims*. Ilexonin A (IA), a component of the Chinese medicine *Ilex pubescens*, has been shown to be neuroprotective during ischemic injury. However, the specific mechanism underlying this neuroprotective effect remains unclear. *Methods*. In this study, we employed a combination of immunofluorescence staining, western blotting, RT-PCR, and behavioral tests, to investigate the molecular mechanisms involved in IA regulation of neuronal proliferation and regeneration after cerebral ischemia and reperfusion in rodents. *Results*. Increases in *β*-catenin protein and LEF1 mRNA and decreases in GSK3*β* protein and Axin mRNA observed in IA-treated compared to control rodents implicated the canonical Wnt pathway as a key signaling mechanism activated by IA treatment. Furthermore, rodents in the IA treatment group showed less neurologic impairment and a corresponding increase in the number of Brdu/nestin and Brdu/NeuN double positive neurons in the parenchymal ischemia tissue following middle cerebral artery occlusion compared to matched controls. *Conclusion*. Altogether, our data indicate that IA can significantly diminish neurological deficits associated with cerebral ischemia reperfusion in rats as a result of increased neuronal survival via modulation of the canonical Wnt pathway.

## 1. Introduction

Ischemic cerebrovascular disease (ICVD) is a multifactorial disease that has high rate of morbidity, mortality, and disability, which has serious impact on human health and the quality of life, and its high cost of treatment brings a heavy burden to the society and family [1]. Arterial stiffness is one of the most important factors in ischemia cardiovascular and cerebrovascular disease, which will be a potential treatment target; multiple studies have shown that treatment with anti-inflammatory agents decreases arterial stiffness [2, 3]. Inflammation participates in the pathological process of cerebral ischemia; anti-inflammation as a neuroprotective mechanism in acute ischemic stroke holds promise in experimental models but it is poor in clinical practice [4]. While recent progress has occurred in the areas of ultra-early thrombolysis [5] and neuronal protection [6] as potential therapies, a lack of effective treatment exists for most ICVD patients owing to the multifactorial pathogenesis [7]. Recent studies have highlighted the presence of neural stem cells (NSCs) in the adult mammalian brain that can promote neuronal function recovery through neurogenesis [8]. A functional consequence of neurogenesis includes formation of new neural pathways/synaptic connections [9], crucial for physiological processes such as learning and memory [10]. As a result of migration, proliferation, and differentiation of NSCs, adult neurogenesis occurs as a compensatory mechanism during pathological processes (such as cerebral ischemia) [11–14]. Thus, targeting the regeneration of damaged neurons seems a promising therapeutic strategy for ICVD; however, the molecular mechanisms underlying neurogenesis are poorly understood, thus serving as a significant therapeutic barrier.

The canonical Wnt/*β*-catenin pathway has been shown to regulate many essential life processes, such as proliferation, differentiation, migration, and apoptosis related to embryonic development, growth, aging, and death [15]. This pathway has been reviewed in detail elsewhere [16]. Briefly, in the presence of Wnt, *β*-catenin combines with the Frizzled (Frz) protein, resulting in the phosphorylation of the Dishevelled protein (Dsh) which can then combine with Axin to depolymerize the degradation complex and prevent *β*-catenin phosphorylation. Dephosphorylated *β*-catenin can either accumulate in the cytoplasm or enter the nucleus and activate downstream factors such as the lymphoid enhancer factor (LEF)/T-cell factor (TCF), resulting in the regulation of the transcription of target genes [17, 18]. On the other hand without Wnt, *β*-catenin/GSK3*β*/Axin/APC/CK1 forms a degradation complex that leads to *β*-catenin phosphorylation, ubiquitination, and degradation by the proteasome. This results in low levels of *β*-catenin and subsequent Wnt pathway inhibition [19–21]. Thus, the stability and accumulation of *β*-catenin in the cytoplasm are key to the canonical Wnt pathway.

An increasing number of herbal Chinese remedies are currently being tested to treat clinical disorders such as ICVD owing to their efficacy in animal models [22]. Ilexonin A (IA) is a pentacyclic triterpene extracted from the herbal Chinese medicine* Ilex pubescens*. IA has been shown to have anti-inflammatory and antithrombotic effects by reducing blood viscosity, increasing the formation of new capillaries, and promoting blood circulation. IA has also been shown to confer neuroprotection after ischemia injury, subsequently improving clinical symptoms and the degree of nerve function defect in patients with a cerebral infarction [23]. Previous studies in our laboratory have demonstrated that IA can inhibit neuronal apoptosis in the periphery of ischemic tissue and activate axon regeneration, assessed by increased Brdu/NeuN double positive cortical cells upregulated expression of endogenous bFGF and GAP-43, respectively, after ischemia reperfusion injury. These functional changes promote neuroprotection and the recovery of neural function after cerebral ischemia [24, 25]. In our current study, we hypothesized that IA promotes nerve regeneration by targeting canonical Wnt signaling pathway activation. Our data shows alterations in *β*-catenin, GSK3*β*, Axin, and LEF1 that correspond to Wnt pathway activation after IA treatment in the peripheral ischemic tissue following ischemia reperfusion. Furthermore, IA promotes neuronal regeneration and an improvement in neurological function, suggesting IA's role as a potential therapeutic for ICVD.

## 2. Materials and Methods

### 2.1. Animals

Adult male Sprague-Dawley rats weighing 250 g (±10 g) were approved by the laboratory animal center of Fujian Medical University (qualified number: SCXK min. 2012-0001). All rats were allowed free access to food and water and maintained at a mean room temperature of 22–25°C on a 12-hour light/dark cycle.

### 2.2. Animal Groups

144 Sprague-Dawley male rats were randomly divided into four different groups: control, sham, model, and IA group. A middle cerebral artery occlusion (MCAO) was carried out on all groups other than the sham group where the middle cerebral artery was not blocked but other surgical procedures were consistent with the model and IA group. Each group was then subdivided (*n* = 9/subgroup) into 4 subgroups for the duration of reperfusion (1 d, 3 d, 7 d, and 14 d). Neurological deficits were evaluated using the Zea Longa scoring method. Rats with a score between 1 and 3 points were divided into successful rats and utilized for the study. If animals died due to surgical procedures or unexpected circumstances, new rats were added to ensure a similar number of rats in each group.

### 2.3. MCAO Surgery

During the surgery, rodents were anesthetized with 10% chloral hydrate administered via intraperitoneal injection. Nylon thread (diameter: 0.26 mm) was provided by Beijing Sunbio Biotech Co., Ltd. A left MCAO was performed using nylon thread (diameter: 0.26 mm, provided by Beijing Sunbio Biotech Co., Ltd.) for 2 h. Reperfusion durations varied ranging between 1 d, 3 d, 7 d, and 14 d.

### 2.4. IA and 5-Bromodeoxyuridine (Brdu) Treatment

Rodents were administered 40 mg/kg IA intraperitoneally (i.p.) immediately after ischemia reperfusion injury and subsequently once a day till the day before they were sacrificed. Control, sham, and model group rodents were administered the same volume of saline for a similar duration. 2*∗*50 mg/kg, twice a day, i.p. injections of Brdu were administered to rodents for 3 consecutive days before death.

### 2.5. Immunofluorescent Cell Staining

Rats were anesthetized with 10% chloral hydrate and transcardially perfused using ice-cold saline, followed by 4% paraformaldehyde solution. Brains were removed and fixed using 4% paraformaldehyde solution for 24 h and then dehydrated by sucrose gradient dehydration (15%, 20%, and 30%) at 4°C. The entire brain was sliced into 8 *μ*m thick sections by a freezing microtome.

*Brdu/Nestin Double-Labeling Immunofluorescence Staining*. Slices were equilibrated at room temperature for 30 min, washed with phosphate buffered saline (PBS, containing 0.1% Triton X-100) (6 × 5 min), incubated at 37°C for 15 min with 2 mol/L HCl, and immediately washed with boric acid (2 × 6 min) and PBS (6 × 5 min). Tissue was then blocked in normal goat serum (ZSGB-BIO) at 37°C for 1 h and incubated with primary antibody against Brdu (mouse, 1 : 200; Sigma) overnight at 4°C and for 2 h in the dark with secondary antibody (Alexa Fluor 488-Conjugated AffiniPure Goat Anti-Mouse IgG, 1 : 100, ZSGB-BIO). After additional washes, slices were incubated with an anti-nestin primary antibody (mouse, 1 : 50; Santa Cruz) and a secondary antibody (Rhodamine-TRITC-Conjugated AffiniPure Goat Anti-Mouse IgG, 1 : 500, ZSGB-BIO). The slices were observed under a fluorescence microscope. Positive cells were evaluated by Imagine Pro Plus 5.0 in ten nonconsecutive high power microscopic fields (200x) for each section.

*Brdu/NeuN Double-Labeling Immunofluorescence Staining*. Staining was performed similar to procedures described above. Primary antibodies used were against Brdu (mouse, 1 : 200; Sigma) and NeuN (rabbit, 1 : 400; Millipore) overnight at 4°C, followed by their respective secondary antibodies (Rhodamine-TRITC-Conjugated AffiniPure Goat Anti-Mouse IgG, 1 : 500, ZSGB-BIO, and Fluorescein-Conjugated AffiniPure Goat Anti-Rabbit IgG, 1 : 500, ZSGB-BIO). Positive cells were evaluated as previously described.

*β-Catenin Immunofluorescence Staining*. Slices were incubated with primary antibody to *β*-catenin (Rabbit mAb, 1 : 100; Cell Signaling) overnight at 4°C and then washed and incubated in the dark at room temperature for 2 h with secondary antibody (Alexa Fluor 488-Conjugated AffiniPure Goat Anti-Rabbit IgG, 1 : 200, ZSGB-BIO). After washing, the nuclei were stained with DAPI (Beyotime) for 5 min. Positive cells were evaluated as previously described.

### 2.6. Western Blot Analysis

Rodents were anesthetized and transcardially perfused as previously described. Peripheral ischemic tissue and hippocampi were homogenized using RIPA buffer containing PMSF (Beyotime). After centrifugation (14000 rpm, 5 min, 4°C), the supernatants were collected and stored at −80°C. 80 *μ*g of total protein was resolved using 10% SDS-PAGE gels, transferred onto nitrocellulose membranes, blocked with Tris-buffered saline containing 0.1% Tween 20 (TBST) and 5% nonfat milk (Cell Signaling) at room temperature for 1 h, and then incubated with primary antibody for *β*-catenin (Rabbit mAb, 1 : 1000; Cell Signaling), GSK3*β* (Rabbit mAb, 1 : 1000; Cell Signaling), or *β*-actin (mouse monoclonal antibody, 1 : 2000; Santa Cruz) overnight at 4°C. After washes with TBST (5 × 10 min), membranes were incubated with secondary antibody, goat anti-rabbit IgG or goat anti-mouse IgG (1 : 6000, ZSGB-BIO), for 2 h at room temperature and visualized using chemiluminescence (ECL western blotting kit, KPL) on Kodak films. The average gray scale of the band was measured using ImageMaster VDS gel imaging and analysis systems, with *β*-actin protein serving as a loading control.

### 2.7. RT-PCR Analysis

Total RNA was extracted with the TRIZOL reagent (Beijing Dingguo Changsheng Biotech Co., Ltd.). The concentration of RNA was detected by spectrophotometry at 260 nm. 4 *μ*g total RNA was reverse transcribed into cDNA using a volume of 20 *μ*L with 1 *μ*L of 10 mM dNTPs (Genview), 1 *μ*L of 50 *μ*M Oligo (dT)18, 0.5 *μ*L of 100 *μ*M random primers, 4 *μ*L of 5x RT buffer, 1 *μ*L of 200 U/*μ*L MMLV reverse transcriptase (TOYOBO), and 8.5 *μ*L DEPC H_2_O. The mixture was incubated for 10 min at 30°C, 60 min at 42°C, 5 min at 99°C, and 5 min at 4°C. For PCR amplification, 1 *μ*L of synthesized cDNA was analyzed in a solution containing 0.5 *μ*L of 10 *μ*M primers, 12.5 *μ*L of 2x Mix, and 11 *μ*L of ddH_2_O. For rat *β*-actin, the primers used were as follows: forward CACCCGCGAGTACAACCTTC and reverse CCCATACCCACCATCACACC; for Axin, forward ACCTCACATTCCTCGCAC and reverse AGAAGGCATTTCCCCATC; and for LEF1, forward AGCCTGTTTATCCCATCACG and reverse TGAGGCTTCACGTGCATTAG. Amplification was implemented using the following protocol for 35 cycles: initial denaturation for 2 min at 94°C, denaturation for 30 sec at 94°C, primer annealing for 30 sec, extension for 30 sec at 72°C, and the last extension at 72°C for 10 min. Annealing temperatures were as follows: *β*-actin, 56°C; Axin, 52°C; LEF1, 55°C. Products were observed on a 2% agarose gel electrophoresis with ethidium bromide for visualization. The average gray scale of the band was measured using one-Dscan image analysis software, with *β*-actin mRNA as a reference.

### 2.8. Statistical Analysis

All data were expressed as the mean ± standard deviation and analyzed by one-way analysis of variance using the SPSS13.0 software package. Data among the groups were analyzed by homogeneity test of variances. Comparison between groups was performed using the LSD test for covariance and the Games-Howell test for heterogeneity of variance. The effect of drug intervention on rat neural function defect score was analyzed by repeated measures analysis of variance. Comparison between two groups was performed using a *t*-test with *P* < 0.05 being set as the limit for statistical significance for all tests.

## 3. Results

### 3.1. IA Treatment Promoted an Improvement of Neurologic Impairment after Ischemia Reperfusion

Neurological deficits were evaluated using the Zea Longa scoring method: 0, no neurological deficit; 1, a slight neurological deficit, failure to extend right forepaw fully; 2, a moderate neurological deficit, circling to the right; 3, a severe neurological deficit, falling to the right; 4, no spontaneous walking and low level of consciousness [26]. Neurological deficits were not observed in the normal and sham groups. However, in the model group and IA treatment group significant neurological deficits were observed 1 d after reperfusion. The neurological deficit score of the IA treatment group was slightly less than the model group but not significantly so. Over time (3 d and 7 d), neurological deficits reduced significantly in the IA-treated group compared with the model group (Table 1).

### 3.2. IA Treatment Correlated with NSCs Proliferation and Differentiation into Neurons in Ischemic Peripheral Tissue after Ischemia Reperfusion

**Figure 1:**
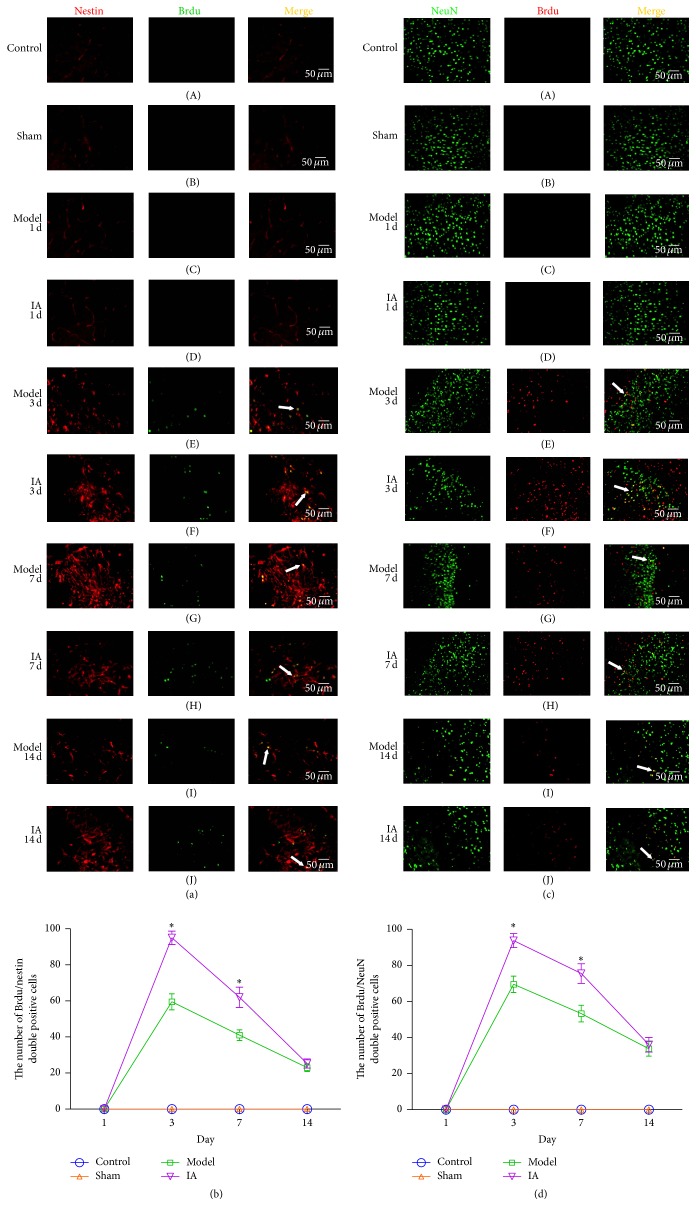
Effect of IA on NSCs proliferation and neuronal regeneration in peripheral ischemic tissue after ischemia reperfusion. (a) Viable NSCs proliferation detected by double-labeling immunofluorescence staining with Brdu (green) and nestin (red). The arrowheads (white) indicated Brdu/nestin double positive cells (orange). (b) Quantitative analysis of Brdu/nestin double positive cells by Imagine Pro Plus 5.0 in ten nonconsecutive high power microscopic fields (200x). ^*∗*^ *P* < 0.05 compared with model group at corresponding point in time. Scale bar: 50 *μ*m. (c) Viable new neurons detected by double-labeling immunofluorescence staining with Brdu (red) and NeuN (green). The arrowheads (white) indicated Brdu/NeuN double positive cells (orange). (d) Quantitative analysis of Brdu/NeuN double positive cells by Imagine Pro Plus 5.0 in ten nonconsecutive high power microscopic fields (200x). ^*∗*^ *P* < 0.05 compared with model group at corresponding point in time. Scale bar: 50 *μ*m.

Brdu is a synthetic thymidine analog that incorporates into DNA during the S phase of cell proliferation cycle, thus serving as a marker of cellular proliferation. Nestin is a characteristic neural stem cell marker and is localized cytoplasmically. NeuN is a protein specific to neurons and is located in the nucleus. Using double staining of Brdu/nestin and Brdu/NeuN double positive cells in the parenchyma of brain ischemic tissue as markers, we observed NSCs proliferation and nerve regeneration. No Brdu/nestin double positive cells were detected in ischemic parenchymal tissue of control and sham rodent at 1 d (Figures 1(A) to (D)). The number of Brdu/nestin double positive cells in the model group significantly increased over time and reached its peak at 3 d (Figure 1(a) (E)), started to decline by 7 d (Figure 1(a) (G)), and continued decreasing in expression at 14 d (Figure 1(a) (I)). Interestingly, the number of Brdu/nestin double positive cells was significantly greater (at 3 and 7 d, Figure 1(a) (F) and (H)) in the IA treatment group compared to the model group (Figure 1(b)), although both groups followed a similar trend over time (peak at 3 d and decline at 7 d).

Anti-Brdu and anti-NeuN antibodies were used to identify Brdu/NeuN double positive cells by double-labeling immunofluorescence in slices from parenchymal ischemic brain tissue. No Brdu/NeuN double positive cells were detected in control and sham groups at 1 d following ischemic reperfusion (Figure 1(c) (A) to (D)). In the model group, Brdu/NeuN double positive cells peaked at 3 d (Figure 1(c) (E)) and began declining at 7 d (Figure 1(c) (G)) while maintaining expression at 14 d (Figure 1(c) (I)). Brdu/NeuN double positive cells were expressed more highly in the treatment group compared to the model group (Figure 1(c) (F) and (H)), with a significant difference being observed at 3 d and 7 d between the treatment and model groups (Figure 1(d)).

### 3.3. IA Treatment Is Associated with the Nuclear Accumulation of Neuronal *β*-Catenin in Ischemic Peripheral Tissue after Ischemia Reperfusion

**Figure 2:**
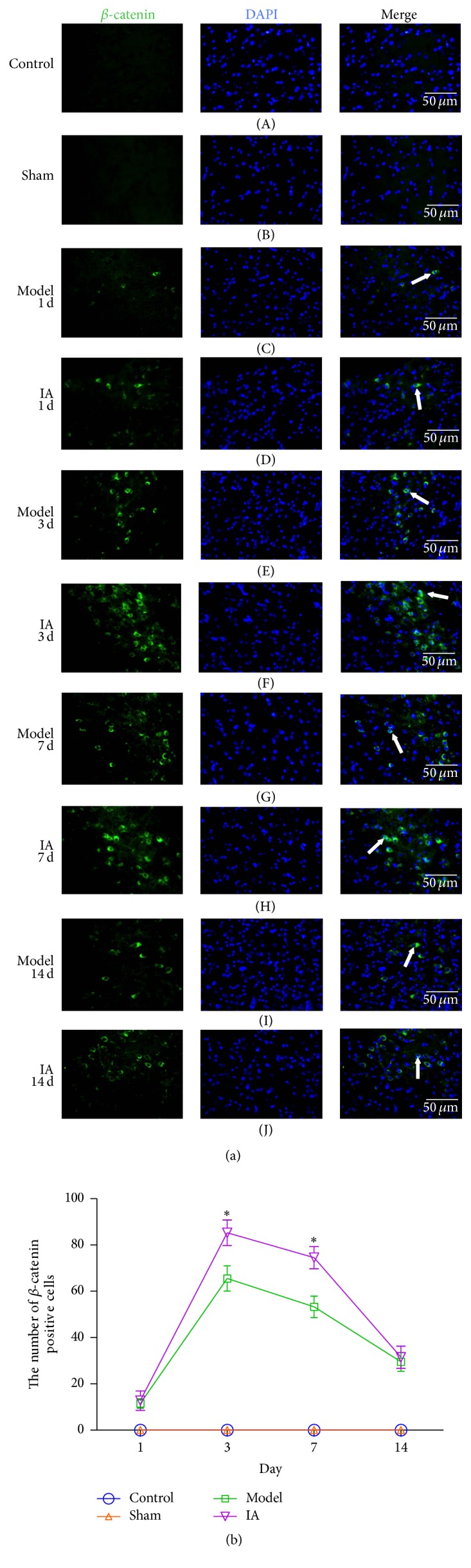
Effect of IA on *β*-catenin expression levels in ischemic peripheral tissue after ischemia reperfusion. (a) *β*-catenin (green) and DAPI (blue) were stained using immunofluorescence techniques. The arrowheads (white) indicated *β*-catenin positive cells (Nattier Blue). (b) Quantitative analysis of *β*-catenin positive cells by Imagine Pro Plus 5.0 in ten nonconsecutive high power microscopic fields (400x). ^*∗*^ *P* < 0.05 compared with model group at corresponding point in time. Scale bar: 50 *μ*m.

Our immunofluorescence results indicate *β*-catenin localization in the cell nucleus of parenchymal ischemic brain tissue owing to significant overlap with DAPI, a nuclear marker. *β*-Catenin was not detected in tissue from control and sham groups (Figure 2(a) (A) and (B)). In tissue obtained from the model group, the number of *β*-catenin positive cells increased at 1 d (Figure 2(a) (C)), peaked at 3 d (Figure 2(a) (E)), and began declining at 7 d (Figure 2(a) (G)). *β*-Catenin expression was still detected at 14 d (Figure 2(a) (I)). A significant difference in *β*-catenin positive cell number was observed at 3 d and 7 d (Figure 2(a) (F) and (H)) between the treatment group and model group. The total number of *β*-catenin positive cells was greater in the treatment group compared to the model group at corresponding point in each time, suggesting increased *β*-catenin expression as a result of IA treatment (Figure 2(b)).

### 3.4. IA Upregulated the Expression Levels of *β*-Catenin Protein and LEF1 mRNA in Ischemic Peripheral Tissue after Ischemia Reperfusion

**Figure 3:**
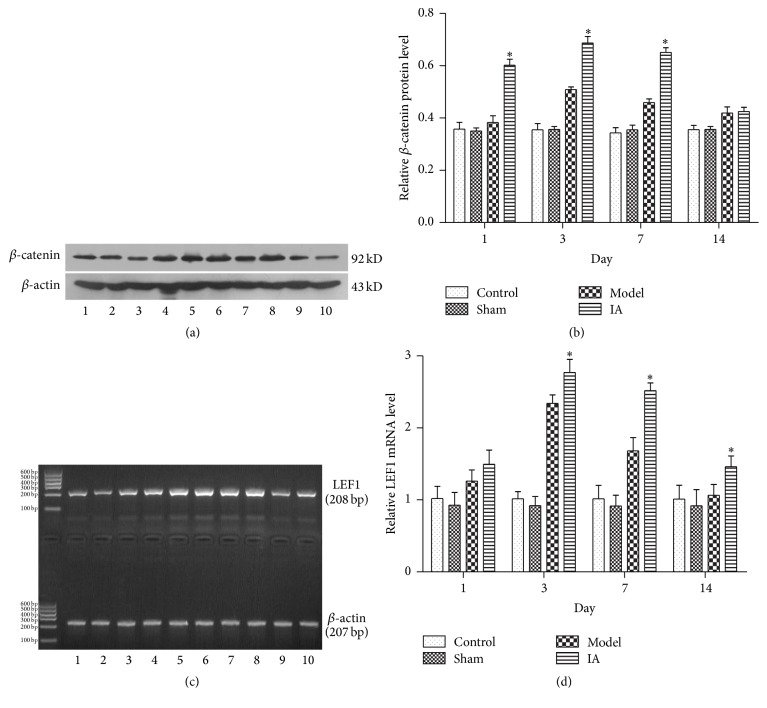
Effect of IA on *β*-catenin protein and LEF1 mRNA expression levels in ischemic peripheral tissue after ischemic reperfusion. *β*-catenin protein and LEF1 mRNA were detected by western blotting or RT-PCR and normalized to *β*-actin as a loading control. Lane 1: control; Lane 2: sham; Lane 3: model 1 d; Lane 4: IA 1 d; Lane 5: model 3 d; Lane 6: IA 3 d; Lane 7: model 7 d; Lane 8: IA 7 d; Lane 9: model 14 d; Lane 10: IA 14 d. (a) *β*-catenin protein increased at 1 d and reached its peak at 3 d and then reduced gradually. The expression of *β*-catenin increased after IA treatment compared with model group at corresponding point in time. (b) Quantitative analysis of *β*-catenin protein. ^*∗*^ *P* < 0.05 compared with model group at corresponding point in time. (c) LEF1 mRNA increased at 1 d and reached its peak at 3 d and then reduced gradually. The expression of LEF1 mRNA increased after IA treatment compared with model group at corresponding point in time. (d) Quantitative analysis of LEF1 mRNA. ^*∗*^ *P* < 0.05 compared with model group at corresponding point in time.

Western blotting detects minimal *β*-catenin protein expression in ischemic peripheral tissue in control and sham groups. *β*-Catenin protein expression in the model group increased at 1 d, peaked at 3 d, and began to decline at 7 d, mirroring the trend seen during immunofluorescence staining (Figure 2). Total *β*-catenin protein was greater in the treatment group compared to the model group at 1 d, 3 d, and 7 d (Figures 3(a) and 3(b)).

Using RT-PCR, an increase in LEF1 mRNA was observed in the treatment group over time, compared to the control, sham, and model groups. LEF1 mRNA was significantly increased compared to the model group at 3 d, 7 d, and 14 d (Figures 3(c) and 3(d); *P* < 0.05).

### 3.5. IA Treatment Resulted in a Decrease in GSK3*β* Protein and Axin mRNA in Ischemic Peripheral Tissue after Ischemia Reperfusion

**Figure 4:**
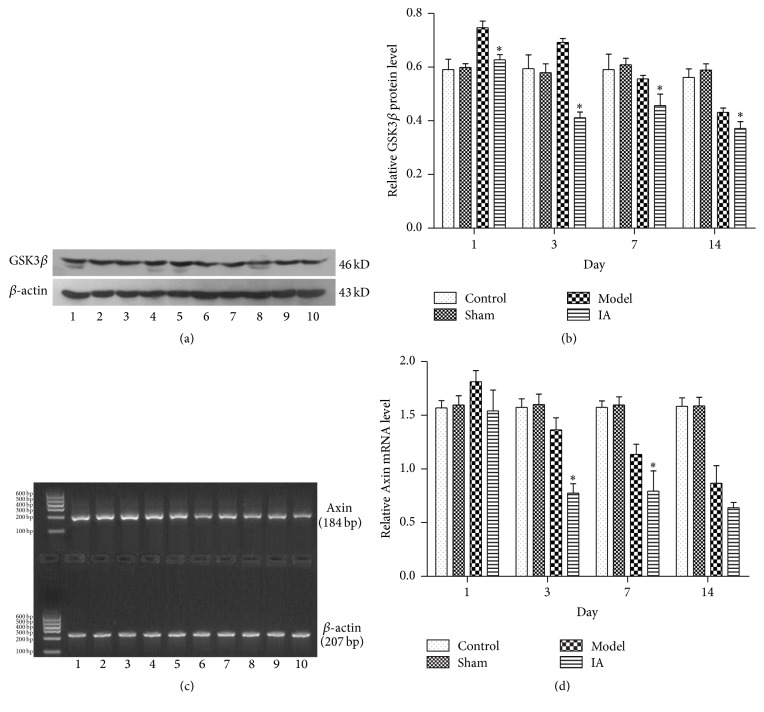
Effect of IA on GSK3*β* protein and Axin mRNA expression detected by western blotting and RT-PCR with *β*-actin protein as a reference. Lane 1: control; Lane 2: sham; Lane 3: model 1 d; Lane 4: IA 1 d; Lane 5: model 3 d; Lane 6: IA 3 d; Lane 7: model 7 d; Lane 8: IA 7 d; Lane 9: model 14 d; Lane 10: IA 14 d. (a) The expression of GSK3*β* declined after IA treatment compared with model group at corresponding point in time. (b) Quantitative analysis of GSK3*β* protein. ^*∗*^ *P* < 0.05 compared with model group at corresponding point in time. (c) The expression of Axin mRNA declined after IA treatment compared with model group at corresponding point in time. (d) Quantitative analysis of Axin mRNA. ^*∗*^ *P* < 0.05 compared with model group at corresponding point in time.

Greater GSK3*β* protein expression was detected in the control, sham, and model groups compared to the treatment group. In the model group, the GSK3*β* protein levels declined over time. GSK3*β* protein was significantly less in the treatment group compared to the model group at all time points tested (1 d, 3 d, 7 d, and 14 d; Figures 4(a) and 4(b)).

Using RT-PCR, decreased Axin mRNA was detected in the treatment group compared to the control, sham, and model groups. This decrease was significant at 3 d and 7 d compared to the model group (Figures 4(c) and 4(d); *P* < 0.05).

### 3.6. Correlating Canonical Wnt Signaling Pathway Activation with Proliferation and Differentiation of NSCs in Ischemic Peripheral Tissue after Ischemia Reperfusion and Treatment with IA

**Figure 5:**
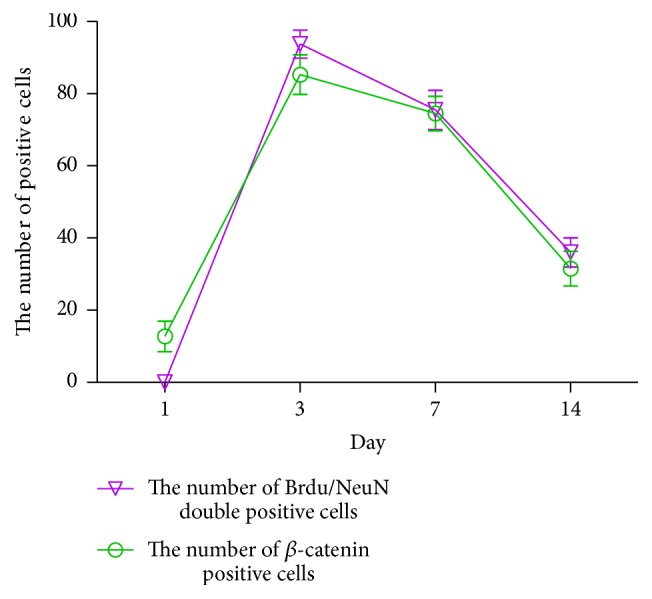
IA treatment group's corresponding trend of Brdu/NeuN double positive cell and *β*-catenin positive cell at corresponding time point after ischemia reperfusion in rats.

Our data thus far suggests that IA treatment after ischemia reperfusion leads to an increase in neuronal proliferation, regeneration, and activation of the Wnt pathway. The shapes of the curves over time for Brdu/NeuN double positive cells and *β*-catenin positive cells after IA treatment were extremely similar, with both curves peaking at 3 d and then gradually declining at time points measured after (Figure 5). We found a significant positive correlation between the two groups at different time points after ischemia reperfusion in rats (*r* = 0.930; *P* = 0.000, <0.01).

## 4. Discussion

During cerebral ischemia, tissue damage occurs in the central region and penumbra of the injury. Neuronal death in the former is an irreversible phenomenon; however, injured neurons in the ischemic penumbra can be restored since a large number of cells can survive the ischemic insult if their blood supply is restored in a timely manner [27]. In recent years, the rise of cerebral vascular intervention therapy helps to improve the prognosis of the acute ischemic stroke patients, but the curative effect about angioplasty and stent implantation is not clear with the guideline from the American Heart Association/American Stroke Association [28]; the effective therapeutic method is needed for the ischemic cerebrovascular disease. Neurotrophic factors such as brain derived neurotrophic factor (BDNF), vascular endothelial growth factor (VEGF), basic fibroblast growth factor (bFGF), and hepatocyte growth factor (HGF) confer neuroprotection by increasing the proliferation and differentiation of endogenous neural stem cells [29–31]; however, an adequate number of neural stem cells are not activated to differentiate into neurons, thus rendering the curative effect of neurotrophic factors incomplete. In recent years, the induction of pluripotent stem cells and cell transplantation* in vivo* has become an important area of research with bone marrow mesenchymal stem cells (BMSCs) serving as important seed cells in the field of stem cell transplantation due to their strong proliferative ability and multidirectional differentiation potential. However, a major problem with BMSCs transplantation for the treatment of cerebral ischemia is the achievement of adequate BMSC migration to the diseased region and subsequent differentiation into nerve cells and survival. NSCs in the SVZ, SGZ, spinal cord, and cerebral cortex in adult mammalian brain have been shown to improve the function of the central nervous system via cell proliferation and differentiation into neurons under certain conditions, such as the sublethal focal cerebral ischemia [32]. Additionally, ischemic studies performed using a middle cerebral artery occlusion model in rodents suggest an increase in cortical neurogenesis (especially in the penumbral region of tissue injury) and increased NSC proliferation in the SVZ and SGZ [15, 33], similar to our study. Chinese traditional medicine has been implicated in the treatment of cerebrovascular disease for a long time. The research of the molecular mechanisms of ischemic cerebrovascular disease helps the prevention and treatment of stroke [34]. The proposed molecular mechanisms of nerve regeneration after cerebral ischemia injury provide a theoretical basis for clinical treatment by Chinese traditional medicine, which have multiple molecular targets. Thus, we chose to study the effects of IA, which has been described to have extensive pharmacological effects such as improving blood circulation, resisting thrombosis and anti-inflammation.

In our study, we found that IA plays an important role in proliferation and the activation of nerve regeneration in parenchymal ischemic brain tissue after injury. Improved neurological functional recovery was associated with these molecular changes upon IA administration. Our results are consistent with Jiang et al., further reinforcing the notion that the IA-dependent increase in neural regeneration under control conditions may be related to the proliferation, migration, and differentiation of endogenous neural stem cells (NSCs) into neurons. However Jiang et al. do not make the mechanism of neuroprotective effect clear; our study investigates the specific mechanism of IA regulation of neuronal proliferation and regeneration. The comprehensive analysis of signaling pathways helps to reveal the mechanism of action of drugs [35].

Presently, a variety of signaling pathways have been implicated in the regulation of nerve regeneration after cerebral ischemia injury, such as cAMP-PKA-CREB signaling, the Notch pathway and the nitric oxide (NO) system, CPG15 gene, MOP receptor protein, GPR40 receptor protein, and the canonical Wnt pathway that involves *β*-catenin. Additionally, the Wnt pathway is a key regulator of the proliferation, migration, and differentiation of NSCs [36], which plays a significant role in cell fate determination in the cerebral cortex and hippocampus [37]. The canonical Wnt pathway also activates the functional recovery after cerebral ischemic injury by promoting nerve regeneration and neuronal survival [38, 39]. Our data implicated canonical Wnt signaling pathway activation in ischemic peripheral tissue at different time points after ischemia reperfusion after IA treatment, which demonstrate that IA promotes nerve regeneration after cerebral ischemia reperfusion accompanied with alterations in the expression of Wnt/*β*-catenin pathway related factors. According to Sommer et al. [40, 41], however, Wnt signaling molecules can produce different phenotypes as a result of different environmental cues and differential expression in nerve cells and various regions of the brain. Moreover, the Wnt pathway can cross talk with a variety of other signaling pathways producing a multitude of biological effects: Noggin/BMP to inhibit the differentiation of NSCs into glial cells [42], the Notch pathway to increase the differentiation of NSCs into neurons, and growth factors such as VEGF, BDNF, Notch, BMP, Shh, and Ephrin to produce effects related to nerve regeneration [43]. In addition there is cross talk between different signaling pathways, such as PI3 kinase and Hedgehog, which share the GSK3*β* protein and play a critical role in signal transduction. For instance, ischemic preconditioning can activate the PI3K/Akt/GSK3*β* pathway, enhance the expression of Akt, increase the expression of p-GSK3*β*, and lead to neuroprotection, then reducing ischemic damage, thus leading to a complex pathogenesis during ischemia reperfusion that must be meticulously studied.

In conclusion, while our experiments provide a strong experimental basis for the use of IA treatment after cerebral ischemia reperfusion injury, further studies utilizing triple immunofluorescence are needed to assess whether all three of our cell markers (Brdu, *β*-catenin, and NeuN) are colocalized and whether new neurons replace the function of necrotic tissue. As we know that inflammation plays an important role in cerebral ischemic injury, further research of pharmacological effects and mechanism of the anti-inflammation action of IA, which is a natural anti-inflammatory agent, will make it more promising for clinic application. In addition, it is clear that the Wnt pathway is thus extremely complex and we must exercise caution when generalizing results associated with numerous studies. Further research is necessary to explore the specific mechanisms activated by IA treatment.

## Figures and Tables

**Table 1:** Neurologic impairment scores (NSS) (*x* ± *s*).

Group	1 d	3 d	7 d	14 d
Control	0	0	0	0
Sham	0	0	0	0
Model	2.889 ± 0.333	2.556 ± 0.527	2.111 ± 0.601	1.111 ± 0.601
IA	2.667 ± 0.500	1.778 ± 0.833^*∗*^	1.333 ± 0.707^*∗*^	0.778 ± 0.441

^*∗*^ *P* < 0.05 compared with model group at corresponding point in time.
